# Conserved structural features of the lncRNA HOTAIR in breast cancer cells

**DOI:** 10.64898/2026.05.18.725451

**Published:** 2026-05-20

**Authors:** Zion R. Perry, Anish Beeram, Logan Lee, Anna Marie Pyle

**Affiliations:** 1 Department of Molecular Biophysics and Biochemistry, Yale University, New Haven, CT 06511, USA; 2 Department of Molecular, Cellular, and Developmental Biology, Yale University, New Haven, CT 06511, USA; 3 Department of Statistics and Data Science, Yale University, New Haven, CT 06511, USA; 4 Department of Chemistry, Yale University, New Haven, CT 06511, USA; 5 Howard Hughes Medical Institute, Chevy Chase, MD 20815, USA

**Keywords:** RNA secondary structure, MarathonRT, SHAPE-MaP, RNA modifications, primate bioinformatics, breast cancer

## Abstract

Long noncoding RNAs (lncRNAs) regulate diverse cellular processes and are frequently implicated in disease, but their functional mechanisms often remain elusive. One such lncRNA, HOTAIR (Hox transcript antisense intergenic RNA), is a ~2.1 kb mammalian transcript whose overexpression promotes invasion and metastasis in breast cancer. However, the mechanisms by which HOTAIR influences gene regulation in cancer are poorly understood. To approach this problem through a structural lens, we determined the full-length *in cellulo* secondary structure of HOTAIR using chemical probing in a metastatic breast cancer cell line. The resulting structure shows that HOTAIR adopts a multidomain architecture and has local structural features unique to the cellular context. Comparison between *in vitro* and *in cellulo* chemical probing identifies regions of differential accessibility that may indicate context-dependent molecular interactions or folding. Conservation analyses further reveal that HOTAIR is conserved across primates with evidence of structural covariation in specific domains. Together, these results provide a roadmap for future mechanistic studies of structure-function relationships in HOTAIR and its contribution to gene regulation in cancer.

## INTRODUCTION

The classical view of RNA as a messenger for protein translation represents only part of RNA’s complex roles in cells. Noncoding RNAs (ncRNAs) participate in many essential cellular processes, and their mis-regulation is associated with numerous human diseases. Long noncoding RNAs (lncRNAs) are a class of ncRNAs longer than 500 nucleotides that are typically transcribed by RNA polymerase II and often capped, spliced, and polyadenylated (Mattick et al. 2023). Since the discovery of the first human lncRNA Xist in 1992 (Brown et al. 1992), studies of lncRNAs have revealed their diverse regulatory mechanisms at all stages of gene expression. LncRNAs may function *in cis* at their locus of transcription, such as through transcriptional interference or enhancer-like activity, or function *in trans* at distant chromatin or cellular locations (Rinn 2014; Kopp and Mendell 2018). In some cases, the underlying DNA locus may function in transcriptional regulation (Engreitz et al. 2016) or the lncRNA may encode for short peptides or proteins (Kawashima et al. 2003; Li et al. 2020). Despite rapid advances in the lncRNA field, the functional mechanisms of most lncRNAs remain poorly understood.

The lncRNA HOX transcript antisense intergenic RNA (HOTAIR) is a prominent example for which we have an incomplete mechanistic understanding. HOTAIR was first discovered by Rinn et al. through microarray sequencing of the *HOX* loci in human fibroblasts, which revealed a transcript antisense to the HOXC locus on chromosome 12 that regulates gene expression *in trans* at the HOXD locus on chromosome 2 (Rinn et al. 2007). Knockdown of HOTAIR reduced histone 3 lysine 27 trimethylation (H3K27me3) and decreased occupancy of the Polycomb Repressive Complex 2 (PRC2) subunit Suz12 at HOXD promoters, implicating HOTAIR in epigenetic silencing. Subsequent studies by Tsai et al. identified the binding sites for PRC2 near the 5’ end of HOTAIR and for another chromatin silencing complex, Lysine-Specific Demethylase 1 (LSD1), near the 3’ end (Tsai et al. 2010). This led to the model of HOTAIR as a molecular scaffold for coordinating chromatin-modifying complexes. However, later studies found that HOTAIR can mediate transcriptional repression independently of PRC2 binding (Portoso et al. 2017), suggesting that recruitment of chromatin modifiers may occur indirectly and after HOTAIR localization to target genes (Brockdorff 2013). Additional studies have since reported interactions between HOTAIR and diverse protein partners (Meredith et al. 2016; Delhaye et al. 2022; Porman et al. 2022), indicating HOTAIR may have multifunctional roles in gene expression. Although HOTAIR clearly participates in epigenetic silencing at some level, the mechanisms of protein recruitment and target gene regulation remain unresolved.

Although HOTAIR plays a normal role in early development (Rinn et al. 2007), its aberrant overexpression has been extensively linked with cancer progression. In breast cancer, HOTAIR is highly overexpressed in metastatic tumors relative to primary tumors and normal tissue (Gupta et al. 2010). Overexpression of HOTAIR in breast cancer cell lines confirmed its invasive influence in cell culture and mouse models, notably in MDA-MB-231 cells, a triple negative and highly invasive breast cancer cell line. Other lncRNAs also exhibit gain of function, oncogenic phenotypes in various cancer pathways, and HOTAIR serves as a one such oncogenic lncRNA for dissecting how RNA-mediated regulatory mechanisms drive tumor progression (Mozdarani et al. 2020; Winkler and Dimitrova 2022). HOTAIR’s oncogenic affects are associated with many cancers, including liver (Yang et al. 2011), colorectal (Kogo et al. 2011), lung (Loewen et al. 2014), and ovarian (Dong and Hui 2016) cancers. Breast cancer serves as a model system to study paradigms of lncRNA dysregulation that may have broader application.

RNA structure is central for regulating its function. Although there are no high-resolution tertiary structures of any lncRNA, secondary structure is a tractable starting point for discovering lncRNA structural modules that may aid functional studies or 3D structure determination of RNA-protein complexes. Chemical probing is an indirect method of determining the secondary structure of RNA. Selective 2’-hydroxyl acylation analyzed by primer extension (SHAPE) reagents modify the 2’OH of the ribose sugar in the backbone proportionate to its flexibility, and generally base-paired nucleotides will be less modified than single-stranded nucleotides (Siegfried et al. 2014; Smola et al. 2015b). This generates a reactivity profile that can be used to improve *in silico* secondary structure prediction algorithms (Reuter and Mathews 2010).

The first information about HOTAIR secondary structure was obtained on a full-length HOTAIR transcript that was transcribed, natively purified *in vitro*, and then probed using SHAPE probing with capillary electrophoresis (Somarowthu et al. 2015). This work suggested that HOTAIR folds into four modular domains, which were proposed to provide platforms for protein interactors such as PRC2 and LSD1. Since then, advances in chemical probing and sequencing techniques have enabled more accurate RNA structure determination by combining chemical probing with mutational profiling and next generation sequencing. This methodology has been further extended to cellular contexts, and improved probes have been developed (Smola and Weeks 2018). Despite these advances, there are few published *in cellulo* secondary structures of lncRNAs with the exception of Xist, MEG3, MALAT1, and SChLAP1 (Smola et al. 2016; Sherpa et al. 2018; McCown et al. 2019; Monroy-Eklund et al. 2023; Falese et al. 2025; Oh et al. 2025), which provide valuable information. Although *in vitro* structures derived from purified and refolded RNA are informative, the *in cellulo* structure is more informative because molecular interactions, chemical modifications, molecular crowding, or other cellular conditions may influence folding. Particularly when carried out in parallel with *in vitro* probing on natively folded transcripts, the method can yield important insights into lncRNA structure and function.

In this study, we determined the first, full-length *in cellulo* secondary structure of HOTAIR in a breast cancer model system using SHAPE-MaP with the probing reagent 2A3 and an MDA-MB-231 HOTAIR overexpression cell line. We also determined a corresponding *in vitro* structure of HOTAIR using SHAPE-MaP with 2A3, enabling direct comparison of structural accessibility and folding in cellular and *in vitro* contexts. The structures reported here can serve as a guide for further mechanistic studies of HOTAIR structure-function relationships. Finally, we built large-scale alignments of HOTAIR homologs from unannotated primate genomes. These alignments revealed both high conservation of HOTAIR within the primate lineage and covarying base pairs within specific structural motifs. Together, this study supports the conservation of functional structural elements within HOTAIR and provides a foundation for investigating how RNA structure contributes to HOTAIR function.

## RESULTS

### Pipeline for structure probing of a long RNA in breast cancer

**Figure 1.**
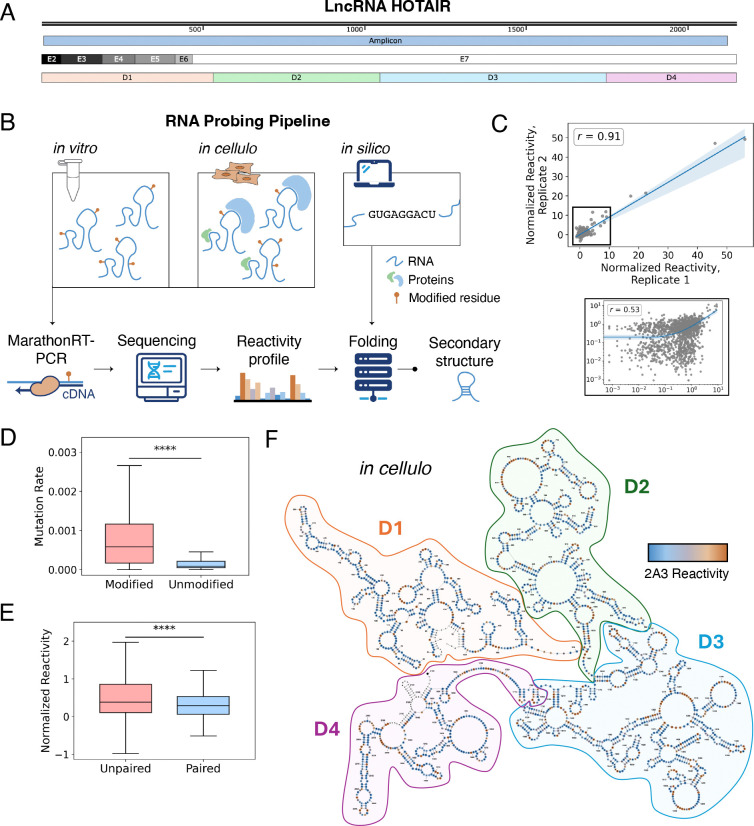
HOTAIR exhibits a complex, multi-domain secondary structure in breast cancer cells. (A) Construct of the lncRNA HOTAIR used in this study. Chemical probing data was captured with a single amplicon spanning 7–2122 nt. Exons are designated from exon 2 to exon 7 (E2-E7), and domains of the *in cellulo* secondary structure are designated as Domain 1 to Domain 4 (D1-D4). Image created using SnapGene. (B) RNA chemical probing pipeline for secondary structure prediction across *in vitro*, *in cellulo*, and *in silico* contexts. Image created using Adobe Stock and Illustrator. (C) Global correlation of normalized reactivities between biological replicates of *in cellulo* SHAPE-MaP on HOTAIR for the full data set or for reactivities < 15. The linear regression line is shown with 95% confidence interval shading. *r*, Pearson’s correlation coefficient. (D) Mutation rates of 2A3-modified samples compared to DMSO-unmodified controls for replicate 1 of *in cellulo* SHAPE-MaP on HOTAIR. Visualized with a box-and-whisker plot. Statistical significance calculated with Mann-Whitney U test, **** = p ≤ 0.0001, *** = p ≤ 0.001, ** = p ≤ 0.01. (E) Normalized reactivities for residues predicted to be unpaired compared to paired in the secondary structure prediction generated from replicate-averaged *in cellulo* SHAPE-MaP reactivities of HOTAIR. Visualized with a box-and-whisker plot. Statistical significance calculated with Mann-Whitney U test, **** = p ≤ 0.0001, *** = p ≤ 0.001, ** = p ≤ 0.01. (F) Secondary structure prediction of HOTAIR using Superfold with replicate-averaged *in cellulo* SHAPE-MaP data. Domains 1–4 are designated as D1-D4. Nucleotides are colored by their normalized reactivity values, orange (≥ 1.0), light orange (≥ 0.75), gray (≥ 0.5), light blue (≥ 0.25), blue ( < 0.25), and white (no data). Structure was visualized with StructureEditor.

We sought to determine an *in cellulo* secondary structure model of full-length HOTAIR using the SHAPE-MaP pipeline (Fig. 1A, B). A major challenge in determining lncRNA structures in cellular contexts is obtaining adequate material for high-quality chemical probing. Unlike ribosomal RNAs, which are present at up to 10^6^ copies per cell, functional lncRNAs are often expressed at 1 to 1,000 copies per cell (Wu et al. 2021). This limits the number of unique cDNA molecules available for sequencing and robust per-nucleotide mutation rate calculations. Additionally, HOTAIR’s size of 2.1 kb requires adequate sequencing reads to be distributed uniformly across the transcript, further reducing per-nucleotide coverage. Together, low copy number and transcript length make *in cellulo* SHAPE-MaP challenging for lncRNAs.

Given HOTAIR’s established role in breast cancer progression, we wanted to perform chemical probing in a relevant breast cancer system. Two breast cancer cell lines commonly used in studies of HOTAIR, MDA-MB-231 and MCF7, expressed 0 to 2 copies per cell, respectively, of endogenous HOTAIR, making them unsuitable for chemical probing (Supplementary Fig. S1A). To overcome the low copy number problem, we chose an MDA-MB-231 HOTAIR overexpression line (MDA-MB-231 HOTAIR) (Porman et al. 2022), which we found to express HOTAIR at ~2,000 copies per cell but only in early-passage cells (Supplementary Fig. S1B). Growing sufficient cells to yield adequate RNA was still challenging due to their slow growth rate and being careful to limit passaging since HOTAIR levels rapidly decrease with increasing passage.

Since this cell line had not been used previously for chemical probing, we validated 2A3 probing conditions using 18S rRNA because it is a highly abundant transcript with a well-understood structure. We probed MDA-MB-231 HOTAIR cells with the SHAPE reagent 2A3, which offers enhanced cell-permeability and accuracy compared to other SHAPE reagents (Marinus et al. 2021). Total cellular RNA was extracted and purified, and a ~1.3 kb region of 18S rRNA was amplified for sequencing and reactivity profile generation via the ShapeMapper2 pipeline (Busan and Weeks 2018). The probing data passed established quality control metrics, including highly correlated reactivity profiles between two biological replicates (Pearson correlation coefficient, r = 0.997; Supplementary Fig. S1C) and significantly higher mutation rates in 2A3-modified 18S rRNA compared to unmodified controls in both replicates (Supplementary Fig. S1D).

After validating chemical probing conditions, the same total cellular RNA was used to amplify a single, ~2.1 kb amplicon spanning 7–2122 nt of HOTAIR. We took advantage of the highly processive reverse transcriptase MarathonRT to allow contiguous amplification of a single sequencing amplicon for *in cellulo* samples, eliminating amplicon-level bias. The overexpression construct used in the MDA-MB-231 HOTAIR cells corresponds to the canonical isoform of HOTAIR, containing exons 2 through 7 (Fig. 1A). Reactivity profiles generated using the SHAPE-MaP pipeline showed high global correlation between two biological replicates (r = 0.91; Fig. 1C). Removing the most reactive nucleotides (normalized reactivity > 15) reduced the correlation to weakly positive (r = 0.53). This is consistent with recent *in cellulo* probing studies of lncRNAs, in which lowly reactive nucleotides show increased noise (Falese et al. 2025). Mutation rates were significantly higher in 2A3-modifed samples compared to unmodified controls in both replicates (Fig. 1D; Supplementary Fig. S1E), indicating that the reactivity data was sufficient for proceeding to secondary structure prediction. The same negative control samples from SHAPE-MaP were further used with MarathonRT in the MRT-ModSeq pipeline to predict candidate modification sites (Tavares et al. 2023). The MDA-MB-231 HOTAIR cell line and technical benefits of MarathonRT thus enabled determination of an *in cellulo* structure and features of HOTAIR in breast cancer.

### Determining the global architecture of HOTAIR in cells

Reactivity values were averaged from both biological replicates and used to constrain secondary structure predictions using Superfold (Reuter and Mathews 2010). As expected, nucleotides predicted to be unpaired showed significantly higher reactivity values compared to nucleotides predicted to be paired (Fig. 1E), indicating that the average structure is consistent with experimental reactivity profiles in addition to the individual replicates (Supplementary Fig. S1F). The secondary structure model revealed that HOTAIR folds into four complex structural domains in breast cancer cells (Fig. 1F). Domain boundaries were defined as regions comprising complete structures separated by stretches of single-stranded nucleotides and was guided by the prior *in vitro* structure of HOTAIR (Supplementary Table S1). Domain 1 includes exons 2 through 6, while exon 7 spans the end of Domain 1 and extends through Domains 2–4 (Fig. 1A).

Notably, the prior *in vitro* study reported that HOTAIR folds into four independent structural domains (Somarowthu et al. 2015). The nucleotide boundaries of Domains 1 and 2 closely match those reported *in vitro*, whereas Domain 3 is longer and more complex *in cellulo*, with a shorter Domain 4. The conservation of overall domain architecture despite the absence of proteins and other cellular factors *in vitro* suggests that HOTAIR folds into a stable structure that is largely determined by its primary sequence. In contrast, local structural differences within individual domains of the *in cellulo* model may reflect cellular influences that contribute to HOTAIR function.

### Identification of highly structured modules in HOTAIR

**Figure 2.**
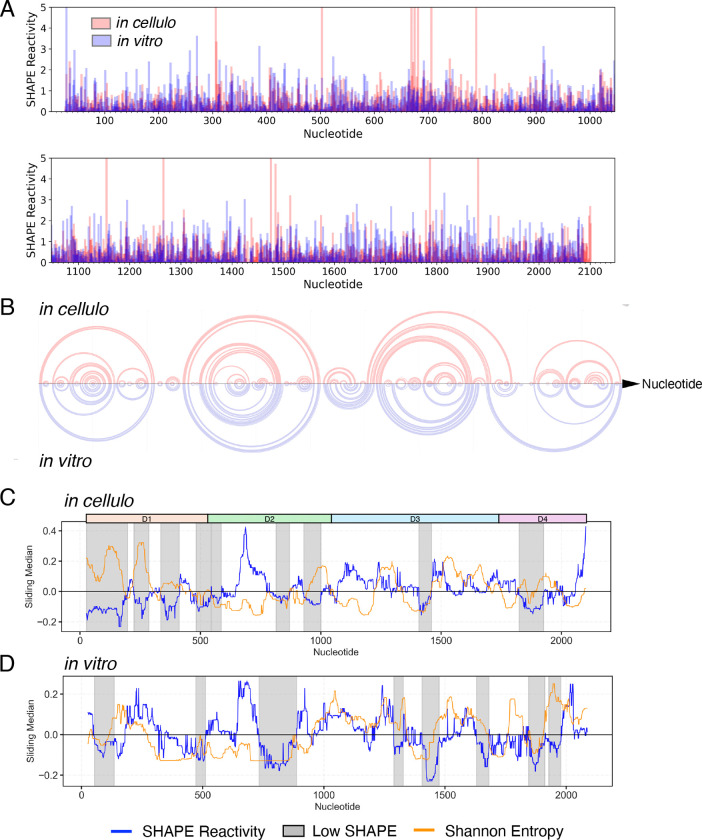
HOTAIR contains highly structured modules across *in cellulo* and *in vitro* contexts. (A) Normalized per-nucleotide reactivities from replicate-averaged *in cellulo* (light red) and *in vitro* (light blue) SHAPE-MaP data. The range was limited to < 5 to enable comparison of the majority of reactivities. (B) Arc plot comparison of the replicate-averaged *in cellulo* and *in vitro* secondary structure predictions. Each arc represents a base pair. Image created with Rchie. (C) Sliding window median analysis of the replicate-averaged reactivity (blue line) and Shannon entropy (orange line) values relative to the global median for *in cellulo* SHAPE-MaP. Gray boxes indicate regions where the SHAPE reactivity is below a specified threshold for at least 40 nt. Domains 1–4 of the *in cellulo* structure are designated as D1-D4. (D) Sliding window median analysis of the replicate-averaged reactivity and Shannon entropy values for *in vitro* SHAPE-MaP. Visualized as in (C).

To enable quantitative comparison of *in cellulo* and *in vitro* models, we determined a new *in vitro* secondary structure of HOTAIR using SHAPE-MaP with 2A3. Full-length HOTAIR was *in vitro* transcribed, semi-natively purified by size-exclusion chromatography, and refolded at 25 mM Mg^2+^, as described previously (Somarowthu et al. 2015). The probing data passed quality control metrics, including high correlation between biological replicates (r = 0.99) and significantly higher mutation rates in the modified compared to unmodified samples (Supplementary Fig. S2A, B). Comparison of replicate-averaged reactivity profiles from *in cellulo* and *in vitro* experiments show overall agreement, with localized regions of increased or suppressed reactivities that may reflect context-dependent differences in RNA accessibility to the probe (Fig. 2A).

Reactivities from individual replicates and replicate-averaged reactivities were used to constrain secondary structure predictions, with higher reactivities mapping to nucleotides predicted to be unpaired in all cases (Supplementary Fig. S2C, D). The domain boundaries of the *in vitro* structure determined in this experiment correspond closely to the previous *in vitro* structure. In this experiment, Domain 3 is predicted to be 115 nt longer, which is closer to that observed *in cellulo* (Supplementary Table S1). Despite areas of differing accessibility (Fig. 2A), there is substantial shared base-pairing between the *in vitro* and *in cellulo* models, particularly in much of Domains 1 and 2, and the overall four-domain architecture is preserved in both contexts (Fig. 2B).

Reactivity and Shannon entropy criteria are commonly used to evaluate the structural stability of motifs within long RNAs. Sliding window median analysis was applied to calculate the replicate-averaged reactivity and entropy profiles relative to the global median (Fig. 2C, D). We identified regions of at least 40 continuous nt that have SHAPE reactivity below the global median (< 0), revealing highly structured modules throughout all four domains. Of these highly structured regions, we can categorize them into those with low Shannon entropy (S < 0), suggesting rigid structures, and those with high Shannon entropy (S > 0), suggesting conformationally heterogeneous structures.

Comparison of the base pairing between *in cellulo* and *in vitro* models reveals domain-level differences in HOTAIR folding. Domain 1 is highly structured in both *in cellulo* and *in vitro* contexts, with highly similar base pairing throughout (Jaccard similarity ~0.8–0.9) (Supplementary Fig. S2E). Part of Domain 1 (~1–350 nt) differs from the *in silico* model showing that our experimental models capture structural information in cells that is different from what is expected from pure thermodynamics. Interestingly, Domain 1 exhibits higher Shannon entropy across much of the domain *in cellulo* (27–182 nt, 224–301 nt, 336–414 nt) compared to *in vitro*.

Domain 2 contains highly structured modules (547–588 nt, 596–645 nt, 788–869 nt) alternating with high reactivity or high Shannon (942–1007 nt) regions *in cellulo*. Both structures have a region of elevated reactivity at ~700 nt, prior to a low SHAPE, low Shannon region.

In contrast, Domains 3 and 4 contain smaller, highly structured and rigid elements *in cellulo* (1408–1457 nt, 2006–2058 nt). Two low SHAPE regions overlap between the *in cellulo* (1408–1457 nt) and *in vitro* (1405–1453 nt) contexts. Although the overall domain architecture is similar in both contexts, HOTAIR contains several structured modules unique to the cellular context, which may be important to the cellular roles of HOTAIR.

### Evidence for protein interaction sites in HOTAIR

**Figure 3.**
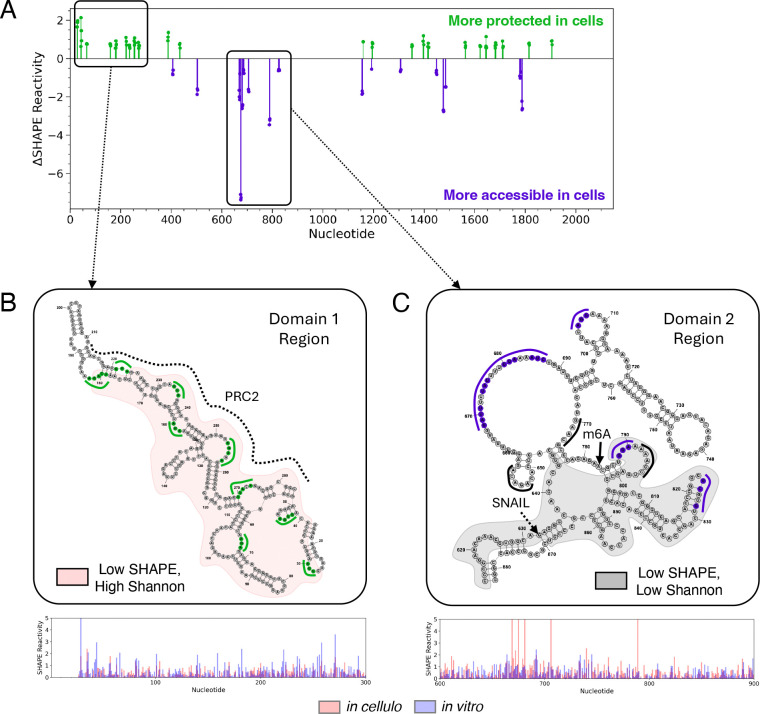
Reactivity differences between *in cellulo* and *in vitro* HOTAIR predict sites of protein protection and structural accessibility. (A) Nucleotides with significant reactivity differences calculated from subtracting replicate-averaged *in cellulo* from *in vitro* reactivities using the deltaSHAPE pipeline. Green indicates sites that are more protected *in cellulo* (ΔSHAPE > 0) and purple indicates sites that are more accessible *in cellulo* (ΔSHAPE < 0). (B) Region of Domain 1 (16–283 nt) with sites of increased protection *in cellulo* (green). This overlaps with a low SHAPE, high Shannon region (light red; 27–182 nt, 224–301 nt) and the minimal PRC2 binding site (black curve; 212–300 nt). Corresponding replicate-averaged reactivity values are shown below the structure. Structure was visualized with StructureEditor. (C) Region of Domain 2 (614–882 nt) with sites of increased accessibility *in cellulo* (purple). This overlaps with a low SHAPE, low Shannon region (gray; 596–645 nt, 788–869 nt), the SNAIL binding site (starting nt indicated with dashed arrow; 632–916 nt), and several m6A sites (black arrow or curve). Corresponding replicate-averaged reactivity values are shown below the structure. Structure was visualized with StructureEditor.

We next identified nucleotide-level differences in reactivities between replicate-averaged *in cellulo* and *in vitro* reactivities using the deltaSHAPE pipeline (Smola et al. 2015a) (Fig. 3A; Supplementary Fig. S3). Positive ΔSHAPE values (> 0) indicate nucleotides that are more protected from modification in cells, which may reflect interactions with cellular factors or structural stabilization. Negative ΔSHAPE values (< 0) indicate nucleotides that are more accessible in cells, which may reflect sites of structural flexibility or unfolding.

Domain 1 contains eleven sites of increased protection and two sites of increased accessibility in cells, each spanning 3–4 nt. The beginning of Domain 1 (16–283 nt) contains a series of highly structured stems that harbor 9 protected sites, which overlaps with the previously determined PRC2 minimal binding region (1–300 nt (Tsai et al. 2010) or 212–300 nt (Wu et al. 2013)) (Fig. 3B). Many of these sites are predicted to be single-stranded, suggesting they would remain structurally accessible for protein interactions. This region also displays relatively high Shannon entropy (Fig. 2C), although it is unclear if protein binding dynamics are the source of this predicted conformational heterogeneity.

A portion of Domain 2 contains a cluster of seven accessible sites (614–882 nt; Fig. 3C) and is flanked by a highly structured, low Shannon entropy region (Fig. 2C). This locally stable structure surrounded by a more accessible loop region may permit protein interactions or tertiary compaction. This region contains a significant portion of the SNAIL binding domain (632–916 nt) and several experimentally mapped N6-methyladenosine (m6A) modification sites (Porman et al. 2022; Garbo et al. 2025). Finally, Domains 3 and 4 have alternating sites of increased protection or accessibility in cells, and seven protected sites overlap with the LSD1 binding site (1500–2146 nt) (Tsai et al. 2010).

Cellular RNA from the control samples in SHAPE-MaP can be further subjected to the MRT-ModSeq pipeline to discover sites of natural modification (Tavares et al. 2023). We identified potential N1-methyladenosine (m1A1243) and N7-methylguanosine (m7G1759) sites in Domain 3 and 4, respectively, which are the most robust sites detectable by MRT-ModSeq (Supplementary Fig. S3B). Sequencing confirmed that these sites were not merely genomic DNA mutations (Supplementary Fig. S3C).

### Conservation and covariation of HOTAIR in primates

Although HOTAIR is present across mammals, its sequence rapidly diverges over evolutionary time (He et al. 2011), so we restricted our analysis to the primate lineage. To better assess conservation but still have sufficient diversity for covariation, we analyzed 190 newly sequenced genomes representing 15 of 16 primate families (Kuderna et al. 2023). Since these genomes are unannotated, we identified candidate loci by searching each genome for homologous sequences to human HOTAIR and then extracting the best-scoring hit for each species (Supplementary Fig. S4A). We found that searching each genome with the full-length transcript of HOTAIR resulted in poor alignment quality, and in many genomes, only recovered exon 7. Instead, we performed three separate searches with exon groups (exons 2–4, exons 4–6, and exon 7) against each genome. We validated that the best-scoring hits for the 3 independent searches were on the same scaffold, which resulted in 179/190 primate species. Of these, we built a multiple sequence alignment for each exon group and removed large gaps (>1,000 nt) not present in human before concatenating the full locus (Supplementary Fig. S4B).

**Figure 4.**
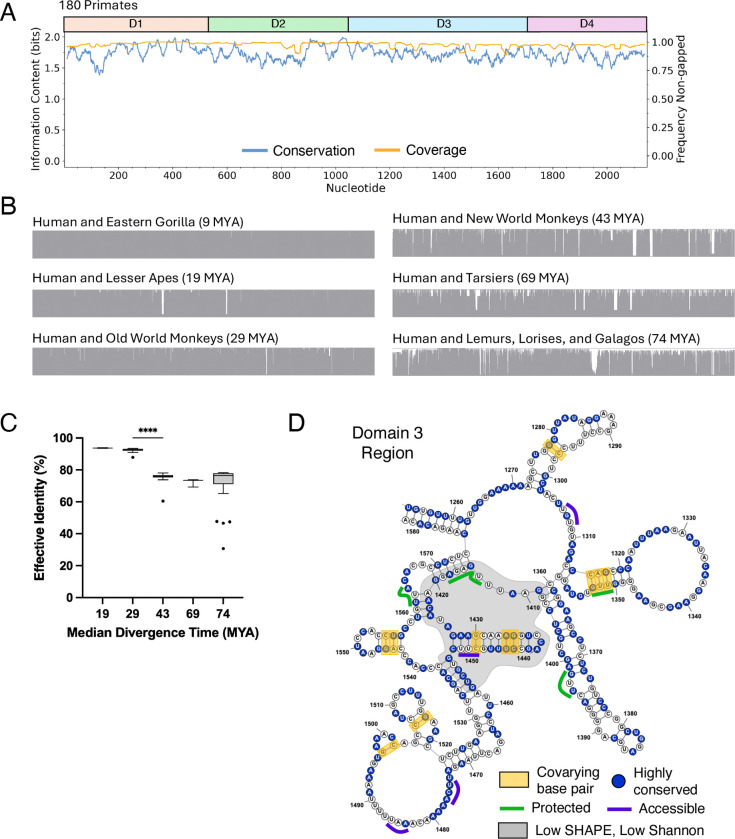
HOTAIR is well-conserved across the primate lineage with evidence for a conserved secondary structure in Domain 3. (A) Rolling average of information content and non-gapped frequency across HOTAIR for a multiple sequence alignment of HOTAIR in 180 primates. Domains 1–4 for the *in cellulo* structure are designated as D1-D4. (B) Conservation histograms of subalignments of each primate taxon extracted from the 180-sequence alignment. Gray bars represent conservation for each column in the alignment. Visualized in UGENE. (C) The pairwise effective identity for each primate compared to human HOTAIR grouped by median divergence time from human in million years ago (MYA). Bar height represents the mean and error bars represent the standard deviation as plotted in GraphPad Prism. Outliers are shown as individual points. Statistical significance calculated with Kruskal-Wallis test with Benjamini Hochberg correction, **** = p ≤ 0.0001. (D) Region of Domain 3 with covarying base pairs (gold), protected sites (green), and accessible sites (purple). Nucleotides with scores > 1.8 bits (the product of information content and non-gapped frequency) from the alignment in (A) are designated as highly conserved. This overlaps with a low SHAPE, low Shannon region (gray; 1408–1457 nt). Structure was visualized with StructureEditor.

Per-nucleotide conservation was assessed across the full alignment by measuring information content and the frequency of non-gapped positions in sliding windows across HOTAIR (Fig. 4A). Regions with high information content and non-gapped frequency reflect positions with high nucleotide conservation and alignment coverage, respectively. From this, we observed that HOTAIR is well-conserved across 179 primates, with a median conservation of 1.88 bits and median coverage of 98.33%. Though there are several peaks of higher conservation, particularly in Domain 1, all 4 domains are equally well-conserved. We observed some insertions in primates (Supplementary Fig. S4B), though these may be misidentified intronic regions rather than true variants.

To assess HOTAIR conservation over evolutionary time, the primate species were grouped based on established primate clades. A subalignment of each clade and human was extracted from the full alignment and gaps present in all species were removed (Fig. 4B). The median percent identity and coverage compared to human for each group was calculated (Supplementary Table S2). High coverage across all primate clades indicates good alignment quality. As expected, the percent identities generally decrease with increasing divergence time from human. The effective identity, which scales the identity by the coverage, was calculated between each species and human. This revealed a significant decrease between primate clades that diverged 29 MYA and 43 MYA, which represents Old and New World monkeys, respectively (Fig. 4C). Even so, the median effective identity of the most ancient divergence group from 74 MYA, the lemurs, lorises, and galagos, has a median effective identity of 76.47%, indicating HOTAIR’s sequence is well-maintained across primates. However, in mouse, which diverged 87 MYA from human, HOTAIR is less conserved with 51.68% identity and 83.19% coverage (Supplementary Fig. S4C; Supplementary Table S2).

This alignment was subsequently filtered to remove similar sequences using a 95% sequence identity threshold, which resulted in 28 representative sequences that were realigned (Supplementary Fig. S4D). This alignment was used to build a maximum likelihood tree to predict the phylogenetic relationships purely based on the HOTAIR sequence (Supplementary Fig. S4E). The predicted tree successfully recapitulates all primate clades present in this alignment. Additionally, the tree can resolve the lemurs from the lorises and galagos, which are distinct infraorders. We also observe accurate branching of different families within the New World monkeys, showing that HOTAIR can capture more subtle divergence patterns. This further validates our identification of the HOTAIR loci and shows that its sequence does not diverge randomly but tracks with known primate phylogenies.

The 95% clustered alignment was then used to assess covariation, which requires sufficient sequence diversity to measure compensatory sequence mutations that preserve base pairing. Covariation analysis on this alignment identified 2 significantly covarying base pairs in Domain 1 and 6 pairs in Domain 3 using the RAFSp statistic (Supplementary Table S3). Additionally, clustering by 99% sequence identity, which resulted in 60 sequences, predicted 5 of the same and 1 additional covarying base pair in Domain 3 and 2 pairs in Domain 4. We next extracted a subalignment of Domain 3 from the 95% clustered alignment. This identified up to 9 covarying base pairs using RAFSp and different window sizes, with 4 unique base pairs not found in the full-length alignment. Of the predicted covarying base pairs, a total of 11 pairs cluster within a discrete structured module of Domain 3, supporting the presence of conserved structures in this domain (Fig. 4D). Interestingly, this region overlaps with several protected and accessible sites in cells (Fig. 3A) and a low SHAPE, low Shannon region that was identified in both the *in cellulo* and *in vitro* contexts (Fig. 2C). These observations suggest that Domain 3 contains a conserved, stably structured element with functional relevance.

Overall, these results show that HOTAIR maintains a global four-domain architecture *in cellulo* with local structural and accessibility differences relative to *in vitro* that correspond to known protein binding sites. In addition, we provide evidence for sequence and structural conservation across the primate lineage in HOTAIR.

## DISCUSSION

In this work, we determined a full-length *in cellulo* secondary structure model of HOTAIR in the triple negative breast cancer cell line MDA-MB-231 using SHAPE-MaP and generated a corresponding *in vitro* structure for quantitative comparison. The structural models show that HOTAIR adopts a four-domain architecture in both contexts but exhibits domain-specific structural differences in cells. In addition, bioinformatic analyses of newly available primate genomes reveal conservation of the HOTAIR sequence and structural conservation of a specific domain. Together, these results provide a framework for more precise structure-function mechanistic studies of HOTAIR in cellular and disease contexts.

Comparison of the *in cellulo* and *in vitro* models revealed both shared and context-dependent structural features that may influence HOTAIR function in cells. The consistency of the four-domain architecture indicates that the global fold is largely encoded by the primary sequence and can form independently of cellular components, such as protein binding partners, chaperones, or molecular crowding. In contrast, structural differences are observed at the individual domain level, suggesting that cellular factors modulate specific structural regions in HOTAIR. For example, there are several low SHAPE reactivity regions that are unique to the *in cellulo* structure. The boundaries of Domains 3 and 4 also differ slightly between contexts, which may be due to the involvement of protein binders, consistent with the fact that these domains overlap with the reported LSD1 binding site (1500–2148 nt) (Tsai et al. 2010). These observations suggest a general folding principle for long RNAs, where the overall architecture is stably encoded by the sequence, but cellular interactions tune local structural features and domain boundaries. Furthermore, single-stranded ‘linker’ regions that separate the discrete structures contained within each domain may provide flexibility for tertiary folding.

Well-structured modules are typically defined as regions with both low SHAPE reactivity and low Shannon entropy across a defined nucleotide window (Siegfried et al. 2014). However, this approach only captures the most static structures within an RNA. By conducting a parallel analysis that identifies regions of low SHAPE reactivity corresponding to high Shannon entropy, we can identify highly structured regions that may exhibit conformational heterogeneity, consistent with recent approaches (Luo et al. 2025). For example, well-paired RNA structures or switches that sample two or more conformational states could be identified through this approach. Structures of this type are predicted in Domain 1 and 2 *in cellulo*, and regions such as this, which are classified as highly structured but are not necessarily rigid, may represent an important class of RNA motifs for which multiple conformations are essential for function.

Beyond structure determination, directly comparing experimental probing data between *in cellulo* and *in vitro* contexts reveals areas of differential reactivity. Generally, these sites are not evenly interspersed on HOTAIR but tend to form ‘hotspots’ of either increased protection or accessibility. Notably, there is a large, protected region in Domain 1 that closely matches the reported PRC2 binding site (1–300 nt) (Tsai et al. 2010; Wu et al. 2013). This region overlaps with a low SHAPE, high Shannon region observed *in cellulo*, and we cannot rule out that protein binding may be confounding these measurements. Low SHAPE reactivity may reflect protection due to protein binding rather than a stable structure, while high Shannon entropy may reflect computational uncertainty in the prediction rather than true conformational heterogeneity.

Conversely, the SNAIL binding domain overlaps with clusters of increased accessibility in Domain 2, suggesting that these types of sites may also facilitate protein binding. The low SHAPE, low Shannon region here may help fold the structure to create a modular platform for protein binding. Interestingly, the mapped m6A sites in this region all correspond to single-stranded regions, making them accessible to modification-interacting enzymes. This region also exhibits distinct base pairing from the *in silico* and *in vitro* models (Supplementary Fig. S2E), making it an interesting structural target unique to the cellular context. We also provide evidence for novel modifications in Domains 3 and 4, which opens new possibilities for HOTAIR-protein regulation. Overall, HOTAIR’s structure is consistent with protein interactions that promote epigenetic silencing and cancer progression. We hope that these findings provide a structural foundation to guide future studies of the HOTAIR-protein interactome in cancer.

Because lncRNAs often diverge rapidly in sequence, restricting conservation analyses to primates allows for better assessment of conservation. Homologous HOTAIR sequences were identified across a diverse dataset of 179 primate genomes, although incomplete assemblies and unannotated genomes hamper identification of the HOTAIR loci in all cases. Additional scaffold validation with neighboring genes on the *HOXC* locus is necessary to further confirm the correct locus identification. Despite these limitations, our alignments reveal substantial sequence conservation across the entire HOTAIR transcript.

Within the primate lineage, we analyzed HOTAIR divergence across evolutionary time from 19 MYA to 74 MYA, with a significant divergence event between 29 MYA and 43 MYA. The 19 MYA and 29 MYA divergence groups represent Hylobatidae (lesser apes) and Cercopithecidae (Old World monkeys), respectively, while the 43 MYA, 69 MYA, and 74 MYA groups represent Platyrrhini (New World monkeys), Tarsiidae (tarsiers), and Strepsirrhini (lemurs, lorises, and galagos). Changes in conservation across these clades may partly reflect the influence of geographic dispersal on HOTAIR sequence divergence.

Furthermore, a representative alignment of HOTAIR can accurately recapitulate known primate phylogenies with family-level resolution, reminiscent of a molecular clock. While multiple rRNA genes have historically been required to define phylogenetic relationships, a single lncRNA achieves comparable resolution here. This suggests that HOTAIR, and potentially other lncRNAs, may be reliable indicators of evolutionary divergence, at least within primates.

Taking advantage of the primate genomes allowed inclusion of novel species and provided sufficient phylogenetic diversity for covariation analysis. We show evidence for covariant base pairs in Domain 3, supporting the functional relevance of a discrete structural module here. This element overlaps with a low SHAPE reactivity and low Shannon entropy region and is close to several protected and accessible sites, all of which indicate potential functional relevance. A few covariant base pairs were predicted in Domains 1 and 4, although our alignments may not have sufficient sequence diversity to detect more covariation. However, by limiting our analysis to primate genomes, we show that HOTAIR is far more conserved than generally appreciated and provide evidence that selective pressure may act on specific structural elements.

A limitation of this study is that HOTAIR overexpression was required to obtain sufficient transcript abundance for probing. Although the overexpression levels are comparable to some patient tumors, the structure presented here may not fully recapitulate native folding in the tumor environment. Future studies that probe endogenous HOTAIR, such as in patient-derived xenograft cell lines (Finlay-Schultz et al. 2020) or primary breast tumor samples (Gupta et al. 2010), would help address this limitation. Additionally, chemical probing reports indirectly on RNA structure and captures an ensemble-averaged snapshot, rather than distinct conformational states. RNAs, like proteins, likely adopt multiple conformations, some of which may guide distinct cellular functions. Future studies in which libraries are constructed from specific subcellular compartments can address this issue. Finally, identification and alignment of lncRNAs across species remains challenging due to frequent insertions and deletions, exon variation, and incomplete genome assemblies, which limits our ability to detect covariation. Addressing these challenges will allow future studies to make critical links between structure and function in HOTAIR and other lncRNA targets.

The *in cellulo* secondary structure model of HOTAIR presented here provides a roadmap for future mechanistic studies. Regions that are highly structured and show increased protection, conservation, or covariation represent promising candidates for investigating protein binding and the role of HOTAIR’s structure in gene regulation and cancer. More broadly, determining structures of lncRNAs in relevant cellular contexts is a key step for connecting structure to function.

## MATERIALS AND METHODS

### Tissue culture

MCF7 (ATCC HTB-22) and MDA-MB-231 (ATCC HTB-26) cells were obtained from the American Type Culture Collection. MDA-MB-231 pBABE-puro HOTAIR overexpression cells (MDA-MB-231 HOTAIR) had been previously generated from parental MDA-MB-231 cells using retroviral transduction as described (Meredith et al. 2016; Porman et al. 2022). Cells were grown in Dulbecco’s Modified Eagle Medium with high glucose and L-glutamine without sodium pyruvate (GenClone, Gibco) with 10% fetal bovine serum (Avantor) and 1% penicillin-streptomycin (Gibco) at 37°C and 5% CO_2_. Media for MDA-MB-231 HOTAIR cells was supplemented with 1 μg/mL puromycin (Gibco).

### *In vitro* transcription and purification for qPCR

#### In vitro transcription.

HOTAIR was transcribed from a pCARs08 plasmid containing the HOTAIR sequence immediately downstream of a T7 promoter. The plasmid had been previously generated as described (Somarowthu et al. 2015). Plasmid was linearized by incubating with SalI restriction enzyme and CutSmart Buffer at 37°C for 16 hrs, followed by enzyme inactivation at 95°C for 5–10 min. Plasmid was purified with the QIAquick Gel Extraction Kit (Qiagen) and added to a 1 mL transcription reaction with transcription buffer (15 mM MgCl2, 40 mM Tris-Cl pH 8, 2 mM spermidine, 10 mM NaCl, 0.01% Triton 100), 10 mM DTT, 4 mM of each NTP at pH 7, RNase OUT (Invitrogen), and 0.08 mg/mL T7 enzyme (purified *in house*). Reaction was incubated at 37°C for 2 hrs, followed by plasmid digestion with 4 μL TURBO DNase (Invitrogen) at 37°C for 30 min and addition of 10 μl of 0.5 M Na_2_EDTA pH 8.5. Transcribed RNA was concentrated to 500 μL and exchanged into 150 mM KCl and 50 mM MOPS pH7 using a 100 kDa Amicon Ultra 0.5 mL filtration column (Amicon).

#### Gel purification.

Urea loading dye (11 M urea, 2% xylene cyanol/bromophenol blue, 50% sucrose, 40 mM Tris-HCl pH 7.5, 0.8 mM Na_2_EDTA pH 8.5 pH 8) was heated at 65°C for 10 min and added to the buffer-exchanged RNA in a 1:1 ratio. An 8 M Urea-5% Acrylamide-Tris-Borate-EDTA (TBE) denaturing gel was cast onto custom-made siliconized gel plates from the Yale University Scientific Glassblowing Laboratory and polymerized with ammonium persulfate and TEMED. The gel was run at 20 W for 30 min prior to loading the RNA sample, which was run at 20–25 W for 3 hours. Gel plates were opened and plastic wrap was laid on top of the gel to flip onto a gel tray. RNA was then visualized under UV light, and the band was extracted with a sterile blade. The RNA was eluted with the ‘crush and soak’ method. Briefly, the gel slice was forced through a 5 ml syringe, 10–12 mL of elution buffer (300 mM NaCl, 10 mM MOPS pH 6, 1 mM Na_2_EDTA pH 8.5) was added, and RNA was eluted at 4°C overnight with shaking. Gel pieces were removed with a filter. 3 M sodium-acetate pH 5.2 was added to precipitate the RNA at −80°C for 2 hrs. To obtain a pellet, the sample was spun at 15,000g for 30 min, washed with 70% EtOH, and spun at 15,000g for another 30 min. The pellet was allowed to dry and redissolved in 1x ME storage buffer (10 mM MOPS pH 6.5, 1 mM Na_2_EDTA pH 8.5). Concentration was determined by Nanodrop at A260.

### Reverse transcription quantitative polymerase chain reaction (RT-qPCR)

#### Reverse transcription (RT).

To generate a standard curve of *in vitro* transcribed HOTAIR, a 10-fold serial dilution of 10^11^ copies/μL to 10^3^ copies/μL was made using the molecular weight calculated from AAT Bioquest (AAT Bioquest 2026). For total cellular RNA samples, cells were dislodged with cell scrapers, centrifuged, and resuspended in Dulbecco’s PBS (GenClone, Gibco). RNA was extracted using 3 parts TRIzol (Invitrogen) to 1 part cell suspension followed by adding chloroform:isoamyl alcohol 24:1 (Sigma) equal to 1/5 TRIzol volume. After centrifuging at 3,000g for 15 min at 4°C, the aqueous RNA phase was removed and precipitated in 75% final EtOH at −80°C for several hours. RNA was pelleted at 15,000g for 30 min at 4°C, air dried, and resuspended in 1x ME buffer. Samples were treated with DNase I (Qiagen) prior to clean-up with the RNeasy kit (Qiagen). For annealing, 1 μg of each RNA sample was combined with 10 mM dNTP Mix (NEB), 2 mM RT primer, and RNase-free water and incubated at 65°C for 5 min in a PCR thermocycler, then placed on ice for 1 min. To the annealing mix, 5x First Strand Synthesis buffer (Invitrogen), 10 mM DTT, 1 μL SUPERase•In RNase inhibitor (Invitrogen), and 1 μL Superscript (SS) II or III (Invitrogen) were added for a final RT reaction volume of 20 μL. Reverse transcription proceeded at 55°C for 50 min for SSIII or 42°C for 50 min for SSII followed by enzyme inactivation at 85°C for 5 min. Template RNA was degraded via alkaline hydrolysis in which 1 μL 3 M KOH was added, the solution was incubated at 95°C for 5 min and 4°C for 2 min, and then 1 μL 3 M HCl was added. The cDNA was cleaned with 1.8x AMPure XP beads (Beckman) according to the manufacturer’s protocol.

#### Quantitative polymerase chain reaction (qPCR).

For each qPCR reaction, 5 μL cleaned cDNA, 2x Light Cycler 480 SYBR Green I Master Mix (Roche), 10 μM forward primer, 10 μM reverse primer, and RNase-free water was combined for a 20 μL reaction. The qPCR plate was covered with a plastic sheet and centrifuged at 2,000g for 2 min. Plates were immediately placed in the qPCR thermocycler and subjected to pre-incubation, amplification (40 cycles), melting curve, and cooling steps.

#### qPCR analysis.

Technical duplicates and/or biological replicates were averaged. The standard curve was plotted and fit to a linear regression line. The slope and y-intercept were used to determine the copies/μl of HOTAIR in cellular samples and converted to copies/cell based on the volume of RNA per dish and the cell count determined using a hemocytometer and Trypan Blue (Sigma). All primers for qPCR are listed in Table of Primers.

### *In cellulo* SHAPE probing

#### Harvesting cells.

MDA-MB-231 HOTAIR cells were grown to 80–90% confluency on two 150 mm dishes per biological replicate. The media was aspirated, the cells were washed with phosphate-buffered saline (PBS), and 10 mL PBS was added to each dish. Cells were dislodged with a cell scraper, pooled, and centrifuged at 1,000g for 5 min at 4°C. Cell pellets were resuspended in 1.5 mL PBS, then evenly split into treated and control fractions.

#### In cellulo SHAPE probing.

Solution of 1 M 2A3 was prepared immediately prior to probing by dissolving 100 mg of 2A3 into 530 μL DMSO (Sigma) and protected from light. For the 2A3-modified fraction, 100 μL of 1 M 2A3 was added per 0.5 mL cell suspension for a final concentration of 100 mM and mixed well. For the DMSO-unmodified control fraction, 100 μL of 100% DMSO was added per 0.5 mL cell suspension and mixed well. Both fractions were incubated at 37°C for 15 minutes protected from light.

#### Total cellular RNA extraction.

To quench the reaction, 3 parts TRIzol (Invitrogen) were added to 1 part cell suspension, mixed well, and incubated for 5 min at room temperature. Next, chloroform:isoamyl alcohol 24:1 (Sigma) equal to 1/5 TRIzol volume was added, mixed well, and incubated for 5 min at room temperature. Samples were centrifuged at 4,700 rpm for 15 min at 4°C, and the aqueous RNA phase was precipitated with 1 μL glycogen per 1 mL aqueous RNA and 75% final EtOH at −20°C for 2–3 days.

#### EtOH precipitation and RNA cleanup.

RNA was pelleted by spinning at 15,000g for 30 min at 4°C. After air drying, each RNA pellet was resuspended in RNase-free water, and in-solution DNase digestion was performed with TURBO DNase buffer and 5 μL TURBO DNase (Invitrogen) at 37°C for 30 min. DNase-treated RNA was purified using the RNeasy kit (Qiagen) and eluted in 1x ME buffer (10 mM MOPS pH 6.5, 1 mM Na_2_EDTA pH 8.5) supplemented with SUPERase•In RNase inhibitor (Invitrogen). Concentration was determined by Nanodrop at A260.

### *In vitro* SHAPE probing

#### In vitro transcription.

HOTAIR pCARs08 plasmid was linearized with SalI-HF restriction enzyme (NEB) and CutSmart Buffer at 37°C for 1 hr with no heat inactivation. Plasmid was precipitated with 1/10 volume of 3 M NaOAc pH 5.5 and 75% EtOH at −20°C for 1 hr. Plasmid was pelleted by spinning at 21,000g for 30 min at 4°C, washed with 70% EtOH, and spun at 21,000g for 5 min. Pellet was allowed to air dry before resuspending in RNase-free water. *In vitro* transcription was performed with transcription buffer (15 mM MgCl2, 40 mM Tris-Cl pH 8, 2 mM spermidine, 10 mM NaCl, 0.01% Triton 100), 10 mM DTT, 2.5 mM of each NTP at pH 7, SUPERase•In RNase inhibitor (Invitrogen), thermostable inorganic pyrophosphatase (NEB), 10 mM MgCl_2_ (Invitrogen) and 0.2 mg/mL T7 enzyme (purified *in house*) for a 1 mL reaction. Reaction was incubated at 30°C for 1.5 hrs, followed by plasmid digestion with 20 μL TURBO DNase (Invitrogen) at 37°C for 10 min and addition of 20 μl of 0.5 M Na_2_EDTA pH 8.5. Transcribed RNA was concentrated and exchanged into filtration buffer (50 mM K-HEPES pH 7.2, 150 mM KCl, 0.1 mM Na_2_EDTA pH 8.5) with 100 kDa Amicon Ultra 0.5 ml filtration columns (Amicon).

#### Semi-native purification and folding.

RNA was semi-natively purified with a fast performance liquid chromatography AKTA using a Sephacryl S-400 column (Cytiva) equilibrated with filtration buffer. RNA elution into 0.5 mL fractions was monitored using the absorbance at A260. The peak fraction was collected, and concentration was determined with Nanodrop at A260. For folding, MgCl_2_ was added to a final concentration of 25 mM and incubated at 37°C for 30 min.

#### In vitro SHAPE probing.

Immediately after folding, the sample was split into 5 μg fractions for each probing condition and replicate. To modified fractions, 2A3 was added for a final concentration of 100 mM. An equivalent volume of DMSO (Sigma) was added to unmodified control fractions. Both fractions were mixed well and incubated at 37°C for 10 min protected from light. To quench the reaction, 1/10 volume of 3 M NaOAc and 75% EtOH were added to each sample and precipitated at −20°C overnight. To pellet RNA, samples were spun at 21,100g for 30 min at 4°C, washed with 70% EtOH, and spun at 21,100g for 5 min at 4°C. Residual EtOH was removed, and the pellet was air dried and resuspended in RNase-free water. Concentration was determined by Nanodrop at A260.

### RT-PCR, library preparation, and sequencing

#### RT-PCR.

All RNA samples from *in cellulo* and *in vitro* SHAPE probing were reverse transcribed with MarathonRT (Guo et al. 2020). Annealing mixes were prepared with 1 μg RNA and 1 μM HOTAIR-specific (or 18S rRNA-specific) primer and annealed at 68°C for 5 min. To each reaction, 2.5x MarathonRT buffer (125 mM Tris-HCl pH 7.5, 500 mM KCl, 12.5 mM DTT, 1.25 mM dNTPs (NEB, Thermo), 2.5 mM MnCl_2_ (Sigma)), MarathonRT (purified *in house*), and glycerol were added and incubated at 42°C for 3 hours, followed by enzyme inactivation at 70°C for 15 min. Single-stranded cDNA was cleaned with either 1.8x RNAClean XP or 1.8x AMPure XP beads (Beckman). Each amplicon was amplified with PCR using Q5 High-Fidelity 2X MasterMix (NEB), 10 μM forward primer, 10 μM reverse primer, and 5 μL cleaned cDNA with touchdown PCR conditions. Annealing temperatures were estimated using SnapGene or the NEB Tm Calculator. PCR products were cleaned with 1.8X AMPure XP beads (Beckman). For *in cellulo* samples, HOTAIR was prepared as a single amplicon (7–2122 nt), while for *in vitro* samples, HOTAIR was tiled with two overlapping amplicons (7–1213 nt and 1010–2122 nt). The control 18S rRNA was prepared as a single amplicon (32–1304 nt) from the same total cellular RNA used for HOTAIR. All primers for SHAPE-MaP are listed in Table of Primers.

#### Library preparation and sequencing.

DNA concentration was measured with the Qubit dsDNA HS Assay Kit (Invitrogen) and diluted to 0.2 ng/μL. Tagmentation and Nextera PCR were carried out according to the Nextera XT DNA Library Prep documentation (Illumina), except at 1/5 the volume. Libraries were purified with 1.8X AMPure XP beads (Beckman) and analyzed on a Bioanalyzer with the High Sensitivity DNA Kit or TapeStation with the HS D5000 ScreenTape Assay (Agilent). Final libraries were adjusted to 4 nM, pooled based on the desired read depth, and adjusted to the final loading concentration required for each kit. Sequencing was performed with the NextSeq 1000/2000 P1 Reagents Kit or the NextSeq 1000/2000 P1 XLEAP-SBS Reagent Kit (Illumina) on a NextSeq 1000/2000.

### Sequencing alignment and structure prediction

Sequencing files were retrieved using the BaseSpace Sequence Hub Downloader and transferred to an HPC cluster. Samples with two amplicons were merged prior to alignment. Paired-end reads were aligned to the full-length HOTAIR sequence, excluding nucleotides up to and including the primer binding sites, using ShapeMapper2 under default parameters (Busan and Weeks 2018). After verifying that samples met sufficient quality control thresholds, the average reactivity per nucleotide was calculated between two biological replicates to create .map files for secondary structure prediction with SuperFold under default parameters (Reuter and Mathews 2010). The *in silico* structure prediction was carried out identically, except the SHAPE reactivity values in the .map file were set to −999 to indicate no experimental data.

### Identification of structured regions

The rolling median of normalized SHAPE reactivity (from ShapeMapper2) and Shannon entropy (from SuperFold) values was calculated in sliding windows of 51 nt around the center nucleotide with step size of 1 nt and truncated edge windows. The global median was subtracted from each rolled value. Typically, low SHAPE regions are defined by reactivity less than the global median (< 0), but we set an arbitrary threshold of < −0.02 to exclude noisy regions near the global median. We identified all low SHAPE regions < −0.02 for a minimum length of 40 nt. We further classified these into low SHAPE and low Shannon entropy (< 0), indicating highly structured regions that adopt a dominant conformation, and low SHAPE and high Shannon entropy (> 0), since these regions are highly structured yet may be conformationally heterogeneous.

### Jaccard similarity calculation

The Jaccard similarity was calculated between each pair of structures in sliding windows of 301 nt around the center nucleotide with step size of 1 nt and truncated edge windows. The Jaccard similarity is defined as the number of shared base pairs (intersection) divided by the total number of unique base pairs (union) in each window.

### ΔSHAPE analysis

Replicate-averaged *in cellulo* SHAPE reactivities were subtracted from replicate-averaged *in vitro* SHAPE reactivities using the deltaSHAPE pipeline under default parameters and statistical thresholds (Smola et al. 2015a). The original deltaSHAPE.py script was first converted to Python 3 compatibility.

### MRT-ModSeq pipeline

The same DMSO-treated control samples from *in cellulo* SHAPE-MaP were further subjected to the MRT-ModSeq pipeline (Tavares et al. 2023). Briefly, reverse transcription was performed with MarathonRT in the presence of 1 mM Mg^2+^ or 2 mM Mn^2+^. Samples were amplified with two overlapping amplicons (7–1114 nt and 1010–2122 nt) and sequenced according to RT-PCR, library preparation, and sequencing. The two amplicons were merged and aligned to HOTAIR with ShapeMapper2, with Mg^2+^ libraries designated as “untreated” and Mn^2+^ libraries designated as “modified.” Machine learning in combination with mutation signature filtering was used to identify the presence of candidate RNA modifications using the published code. Only modifications present in three biological replicates are reported. Genomic DNA (gDNA) from MDA-MB-231 HOTAIR cells was extracted with the Monarch Spin gDNA Extraction Kit. A specific region of the gDNA containing potential modifications was amplified with PCR (see Table of Primers) and samples were sent for Sanger sequencing. Other modifications were identified, including an average of 34 candidate pseudouridine (Ψ) sites per replicate, but these sites tend to have high false positive rates, and only 8 Ψ sites were present in at least two biological replicates. Furthermore, the sequences surrounding the candidate Ψ sites did not match any known pseudouridine synthase recognition motifs, so these modifications were discarded.

### HOTAIR annotation pipeline

#### Exon-aware searches of unannotated genomes.

Assembled primate genomes were downloaded from the European Nucleotide Archive (Accession PRJEB67744), previously deposited by Kuderna et al (Kuderna et al. 2023). For each genome, the tool Exonerate (version 2.4.0) with the est2genome model (Slater and Birney 2005) was used to find potential matches to the given query sequence. Since searches with full-length human HOTAIR (hHOTAIR) returned sequences with poor coverage, three independent searches were conducted with exons 2–4 (288 nt), exons 4–6 (279 nt), and exon 7 (1683 nt). Multiple exons were combined where necessary to reduce false positives from searching with short exons. For each search, the hit with the best alignment score was extracted and converted to RNA using a custom script. Due to the computational requirements, searches were conducted in parallel on a high-performance computing cluster.

#### Scaffold validation and locus construction.

The scaffold corresponding to all three hits per species was cross-checked to confirm the hits were present on the same scaffold. This served as initial validation that our searches produced hits from the same genomic locus. Only 11 species failed this check and were removed from all downstream steps for a final set of 179 primate sequences. The final set of sequences and hHOTAIR (180 sequences total) were aligned using MAFFT (version 7.526, --auto flag) (Katoh and Standley 2013). Inspection of these initial multiple sequence alignments revealed that human exons 2–6 successfully aligned across primates but were separated by large intervening regions (>1,000 nt) absent in humans. These may represent introns that were misidentified by Exonerate, and these regions were removed from each alignment. Finally, the three separate alignments were concatenated to generate the full locus.

### Conservation and evolutionary divergence analysis

The Esl-alistat package in HMMER (version 3.4, http://hmmer.org/) was used to calculate per-column information content in bits and non-gapped frequency in an input alignment. Per-nucleotide scores for hHOTAIR were extracted, and the rolling average was plotted in sliding windows of 21 nt around the center nucleotide with step size of 1 nt. For pairwise conservation analysis, the percent identity and coverage of each species compared to human was calculated using the NCBI MSA Viewer (version 1.26.0) after setting hHOTAIR as the anchor sequence. We define ‘effective identity’ as the product of percent identity and coverage to account for sequences with lower coverage. For evolutionary divergence analysis, each primate was binned by its median pairwise divergence time from human, which was retrieved from TimeTree (Kumar et al. 2022). For divergence bins with at least 3 species, the distribution of effective identities was analyzed. Subalignments of each primate taxon and hHOTAIR were extracted from the 180-species multiple sequence alignment, and gaps present in all species were removed. To compare outside the primate lineage, hHOTAIR was aligned to annotated mouse HOTAIR (NCBI Reference Sequence NR_047528.1) using Clustal Omega (Madeira et al. 2024), and percent identity and coverage were calculated with the NCBI MSA Viewer. All alignments and conservation histograms were visualized with UGENE (version 52.0).

### Clustering and phylogeny analysis

To reduce redundancy from similar sequences, the full alignment (180 sequences) was filtered by 95, 98, or 99% sequence identity (-c flag) using CD-HIT-EST (version 4.8.1) with a word size of 8 (-n flag) (Li and Godzik 2006; Fu et al. 2012). This resulted in 28, 41, and 60 sequences, respectively. After ensuring hHOTAIR was included as the representative sequence of its cluster, they were realigned with MAFFT (version 7.526, --auto flag). Clustered alignments were used for covariation analysis, as described below. The phylogenetic tree was constructed from the 95% clustered alignment using the PhyML Maximum Likelihood method in UGENE (version 52.0). The General Time Reversible (GTR+Γ) substitution model with 4 substitution rate categories was used to allow for unequal base frequencies, variable substitution rates, and rate variation across sites. Branch support was assessed with 1,000 bootstrap replicates in UGENE. The tree was unrooted and visualized in MEGA with the radial layout (version 12.1.1).

### Covariation analysis

Covarying base pairs were identified using R-scape (version 0.2.1) installed from Bioconda (https://anaconda.org/bioconda/rscape/files/manage?page=2). The RAFSp statistical test was selected for its ability to detect subtle covariation in lncRNA alignments (Tavares et al. 2019). Due to the length of HOTAIR, covariation was assessed in sliding windows of 300 or 500 nt (--window) with step size of 100 nt (--slide). Input alignments were first converted to Stockholm format using the Esl-reformat package in HMMER (version 3.4, http://hmmer.org/). The replicate-averaged *in cellulo* secondary structure was added to the alignment, with gaps inserted as single-stranded positions corresponding to the gapped hHOTAIR sequence. Nucleotide positions output by R-scape were converted to positions in the ungapped hHOTAIR sequence.

### Plotting and statistical analysis

Unless otherwise noted, all plots were generated using Matplotlib or Seaborn in Jupyter Notebook or JupyterLab. For box-and-whisker plots, boxes indicate the interquartile range (IQR), the center line is the median, and the whiskers extend to the farthest data point within 1.5xIQR. Statistical significance was calculated with a two-tailed Mann-Whitney U test or an unpaired, two-tailed Student’s t-test, where **** = p ≤ 0.0001, *** = p ≤ 0.001, ** = p ≤ 0.01, and * = p ≤ 0.05.

## Supplementary Material

Supplement 1

Supplement 2

Supplement 3

Supplement 4

Supplement 5

Supplement 6

Supplement 7

## Data Availability

Raw and processed sequencing data from SHAPE-MaP and MRT-ModSeq have been deposited in NCBI’s Gene Expression Omnibus (Edgar et al. 2002) (accession number pending). Processed SHAPE-MaP, deltaSHAPE, MRT-ModSeq, and bioinformatics data are available as Supplemental Data files. Structure files, reference sequences, multiple sequence alignment files, and analysis code is available on GitHub at https://github.com/pylelab/HOTAIR.
