# Supplementary material for: Conserved structural features of the lncRNA HOTAIR in breast cancer cells
Source: bioRxiv. 2026 May 20:2026.05.18.725451. Preprint. [Version 1] doi: 10.64898/2026.05.18.725451 (PMC13228351; doi:10.64898/2026.05.18.725451)
Supplement: Supplement 1 [file media-1.pdf]

Supplemental Material:

Conserved structural features of the lncRNA HOTAIR in breast cancer cells

Zion R. Perry[1], Anish Beeram[2], Logan Lee[1,3], Anna Marie Pyle[2,4,5]

[1]Department of Molecular Biophysics and Biochemistry, Yale University, New Haven, CT 06511 USA

[2]Department of Molecular, Cellular, and Developmental Biology, Yale University, New Haven, CT 06511, USA

[3]Department of Statistics and Data Science, Yale University, New Haven, CT 06511, USA

[4]Department of Chemistry, Yale University, New Haven, CT 06511, USA

[5]Howard Hughes Medical Institute, Chevy Chase, MD 20815, USA

Supplementary Materials and Methods……………………………………………………2-5

Human HOTAIR Sequence

Table of Primers

Supplementary Figures……………………………….……….………….…………...6-12

Supplementary Figure S1. Validation of chemical probing of HOTAIR and 18S rRNA in breast cancer cells.

Supplementary Figure S2. Determination of *in vitro* secondary structure of HOTAIR, structured modules *in cellulo*, and comparison to *in silico* prediction.

Supplementary Figure S3. Differential reactivity sites and evidence for chemical modifications on *in cellulo* HOTAIR.

Supplementary Figure S4. HOTAIR sequence annotation and conservation in primates.

Supplementary Tables………………………………………………………….........13-15

Supplementary Table S1. Domain boundaries for *in cellulo*, *in vitro*, and *in silico* HOTAIR secondary structure models.

Supplementary Table S2. Conservation of HOTAIR between human and different primate taxa and mouse.

Supplementary Table S3. Covarying base pairs in the *in cellulo* structure detected in primate alignments of HOTAIR.

Supplementary Materials and Methods

Human HOTAIR Sequence

The human HOTAIR sequence used in this study comprises nucleotides 1-2148 (excluding the polyA tail) from GenBank Accession DQ926657.1, deposited by Rinn et al. (Rinn et al. 2007). Exon boundaries were determined from NCBI Reference Sequence NR_003716.4; note that this reference is missing the first 5 nt and has a slightly longer final exon compared to the original HOTAIR sequence. Exon 2, 1-60 nt; Exon 3, 61-186 nt; Exon 4, 187-288 nt; Exon 5, 289-412 nt; Exon 6, 413-465 nt; Exon 7, 466-2148 nt. Exon 1 is present in some annotated isoforms and is separated by a relatively long intron.

GACUCGCCUGUGCUCUGGAGCUUGAUCCGAAAGCUUCCACAGUGAGGACUGCUC

CGUGGGGGUAAGAGAGCACCAGGCACUGAGGCCUGGGAGUUCCACAGACCAACA

CCCCUGCUCCUGGCGGCUCCCACCCGGGGCUUAGACCCUCAGGUCCCUAAUAUCC

CGGAGGUGCUCUCAAUCAGAAAGGUCCUGCUCCGCUUCGCAGUGGAAUGGAACG

GAUUUAGAAGCCUGCAGUAGGGGAGUGGGGAGUGGAGAGAGGGAGCCCAGAGU

UACAGACGGCGGCGAGAGGAAGGAGGGGCGUCUUUAUUUUUUUAAGGCCCCAAA

GAGUCUGAUGUUUACAAGACCAGAAAUGCCACGGCCGCGUCCUGGCAGAGAAAA

GGCUGAAAUGGAGGACCGGCGCCUUCCUUAUAAGUAUGCACAUUGGCGAGAGAA

UUAAGUGCUGCAACCUAAACCAGCAAUUACACCCAAGCUCGUUGGGGCCUAAGC

CAGUACCGACCUGGUAGAAAAAGCAACCACGAAGCUAGAGAGAGAGCCAGAGGA

GGGAAGAGAGCGCCAGACGAAGGUGAAAGCGAACCACGCAGAGAAAUGCAGGCA

AGGGAGCAAGGCGGCAGUUCCCGGAACAAACGUGGCAGAGGGCAAGACGGGCAC

UCACAGACAGAGGUUUAUGUAUUUUUAUUUUUUAAAAUCUGAUUUGGUGUUCC

AUGAGGAAAAGGGAAAAUCUAGGGAACGGGAGUACAGAGAGAAUAAUCCGGGU
CCUAGCUCGCCACAUGAACGCCCAGAGAACGCUGGAAAAACCUGAGCGGGUGCC
GGGGCAGCACCCGGCUCGGGUCAGCCACUGCCCCACACCGGGCCCACCAAGCCCC
GCCCCUCGCGGCCACCGGGGCUUCCUUGCUCUUCUUAUCAUCUCCAUCUUUAUG
AUGAGGCUUGUUAACAAGACCAGAGAGCUGGCCAAGCACCUCUAUCUCAGCCGC
GCCCGCUCAGCCGAGCAGCGGUCGGUGGGGGACUGGGAGGCGCUAAUUAAUUG
AUUCCUUUGGACUGUAAAAUAUGGCGGCGUCUACACGGAACCCAUGGACUCAUA
AACAAUAUAUCUGUUGGGCGUGAGUGCACUGUCUCUCAAAUAAUUUUUCCAUAG
GCAAAUGUCAGAGGGUUCUGGAUUUUUAGUUGCUAAGGAAAGAUCCAAAUGGG
ACCAAUUUUAGGAGGCCCAAACAGAGUCCGUUCAGUGUCAGAAAAUGCUUCCCC
AAAGGGUUGGCAGUGUGUUUUGUUGGAAAAAAGCUUGGGUUAUAGGAAAGCCU
UUCCCUGCUACUUGUGUAGACCCAGCCCAAUUUAAGAAUUACAAGGAAGCGAAG
GGGUUGUGUAGGCCGGAAGCCUCUCUGUCCCGGCUGGAUGCAGGGGACUUGAGC
UGCUCCGGAAUUUGAGAGGAACAUAGAAGCAAAGGUCCAGCCUUUGCUUCGUGC
UGAUUCCUAGACUUAAGAUUCAAAAACAAAUUUUUAAAAGUGAAACCAGCCCUA
GCCUUUGGAAGCUCUUGAAGGUUCAGCACCCACCCAGGAAUCCACCUGCCUGUU
ACACGCCUCUCCAAGACACAGUGGCACCGCUUUUCUAACUGGCAGCACAGAGCA
ACUCUAUAAUAUGCUUAUAUUAGGUCUAGAAGAAUGCAUCUUGAGACACAUGG
GUAACCUAAUUAUAUAAUGCUUGUUCCAUACAGGAGUGAUUAUGCAGUGGGAC
CCUGCUGCAAACGGGACUUUGCACUCUAAAUAUAGGCCCCAGCUUGGGACAAAA
GUUGCAGUAGAAAAAUAGACAUAGGAGAACACUUAAAUAAGUGAUGCAUGUAG
ACACAGAAGGGGUAUUUAAAAGACAGAAAUAAUAGAAGUACAGAAGAACAGAA
AAAAAAUCAGCAGAUGGAGAUUACCAUUCCCAAUGCCUGAACUUCCUCCUGCUA

UUAAGAUUGCUAGAGAAUUGUGUCUUAAACAGUUCAUGAACCCAGAAGAACGC
AAUUUCAAUGUAUUUAGUACACACACAGUAUGUAUAUAAACACAACUCACAGAA
UAUAUUUUCCAUACAUUGGGUAGGUAUGCACUUUGUGUAUAUAUAAUAAUGUA
UUUUCCAUGCAGUUUUAAAAUGUAGAUAUAUUAAUAUCUGGAUGCAUUUUC

Table of Primers

**Primer**	**Sequence (5' to 3')**
**HOTAIR qPCR**	
qPCR RT	TTCTACCAGGTCGGTACTGG
qPCR F (Porman et al. 2022)	TCTGGAGCTTGATCCGAAAG
qPCR R (Porman et al. 2022)	GGTGTTGGTCTGTGGAACT
**HOTAIR *in vitro* SHAPE-MaP**	
HOTAIR SHAPE-MaP RT Amp 1	CGGACTCTGTTTGGGCCTCCT
HOTAIR SHAPE-MaP PCR F Amp 1	CCTGTGCTCTGGAGCTTGAT
HOTAIR SHAPE-MaP PCR R Amp 1	ACTCTGTTTGGGCCTCCT
HOTAIR SHAPE-MaP RT Amp 2	ATCTACATTTTAAAACTGC
HOTAIR SHAPE-MaP PCR F Amp 2	AGGCGCTAATTAATTGATTCC
HOTAIR SHAPE-MaP PCR R, Amp 2	CTACATTTTAAAACTGCATGG
**HOTAIR *in cellulo* SHAPE-MaP**	
HOTAIR SHAPE-MaP RT	ATCTACATTTTAAAACTGC
HOTAIR SHAPE-MaP PCR F	CCTGTGCTCTGGAGCTTGAT
HOTAIR SHAPE-MaP PCR R	CTACATTTTAAAACTGCATGG
**MRTModSeq**	

ModSeq RT Amp 1	TGAGAGACAGTGCACTCAC
ModSeq PCR F Amp 1	CCTGTGCTCTGGAGCTTGAT
ModSeq PCR R Amp 1	GAGACAGTGCACTCACGC
ModSeq RT Amp 2	ATCTACATTTTAAAACTGC
ModSeq PCR F Amp 2	AGGCGCTAATTAATTGATTCC
ModSeq PCR R Amp 2	CTACATTTTAAAACTGCATGG
**18S rRNA SHAPE-MaP**	
18S SHAPE-MaP RT	GAAAGAGCTATCAATCTGTC
18S SHAPE-MaP PCR F	TGTCTCAAAGATTAAGCCATGC
18S SHAPE-MaP PCR R	AGCTATCAATCTGTCAATCC
**gDNA Sequencing**	
PCR Forward gDNA	TGTAAAACGACGGCCAGTGGAAAGATCC AAATGGGACC
PCR Reverse gDNA	CAGGAAACAGCTATGACCAATTGCGTTC TTCTGGGTTC

RT = reverse transcription, F = forward, R = reverse, Amp = amplicon

Supplementary Figures

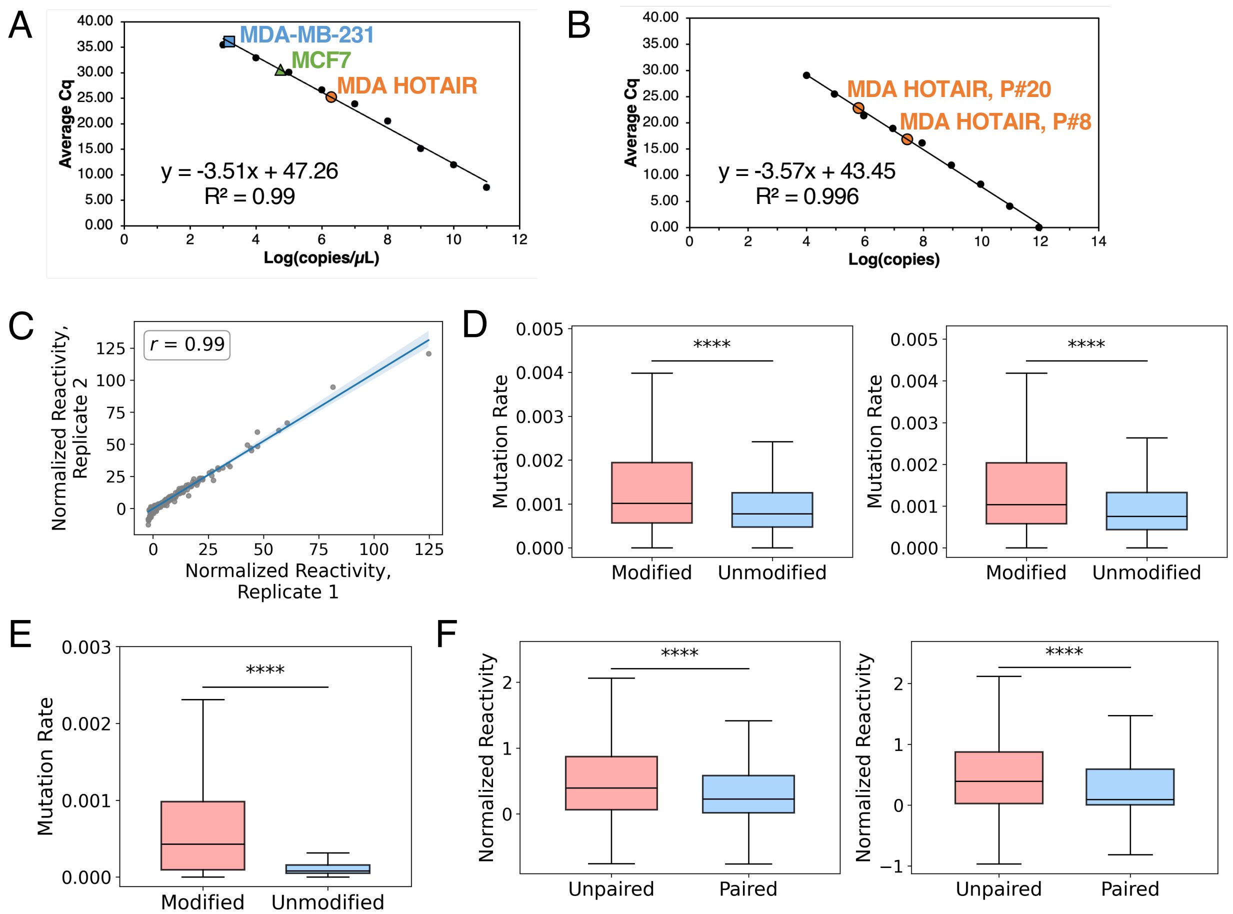

Supplementary Figure S1. Validation of chemical probing of HOTAIR and 18S rRNA in breast cancer cells. (A) Expression of HOTAIR in MDA-MB-231, MCF7, and MDA-MB-231 HOTAIR cell lines determined by absolute RT-qPCR. I*n vitro* transcribed HOTAIR was used to generate a standard curve and fitted to a linear regression line to determine copy number. Quantification cycle (Cq) is average of two biological replicates done in duplicate. Plotted in Microsoft Excel. (B) Expression of HOTAIR in MDA-MB-231 HOTAIR lines at two different passage numbers (P#) determined by absolute RT-qPCR. I*n vitro* transcribed HOTAIR was used to generate a standard curve and fitted to a linear regression line to determine copy number. Cq measured in duplicate. Plotted in Microsoft Excel. (C) Global correlation of normalized

reactivities between biological replicates of *in cellulo* SHAPE-MaP on18S rRNA. The linear regression line is shown with 95% confidence interval shading. *r*, Pearson's correlation coefficient. (D) Mutation rates of 2A3-modified samples compared to DMSO-unmodified controls for two replicates of *in cellulo* SHAPE-MaP on 18S rRNA. Visualized with a box-and-whisker plot. Statistical significance calculated with Mann-Whitney U test, **** = $p \leq 0.0001$, *** = $p \leq 0.001$, ** = $p \leq 0.01$. (E) Mutation rates of 2A3-modified samples compared to DMSO-unmodified controls for replicate 2 of *in cellulo* SHAPE-MaP on HOTAIR. Visualized with a box-and-whisker plot. Statistical significance calculated with Mann-Whitney U test, **** = $p \leq 0.0001$, *** = $p \leq 0.001$, ** = $p \leq 0.01$. (F) Normalized reactivities for residues predicted to be unpaired compared to paired in the secondary structure prediction generated from *in cellulo* SHAPE-MaP reactivities of HOTAIR for replicate 1 (left) and replicate 2 (right). Visualized with a box-and-whisker plot. Statistical significance calculated with Mann-Whitney U test, **** = $p \leq 0.0001$, *** = $p \leq 0.001$, ** = $p \leq 0.01$.

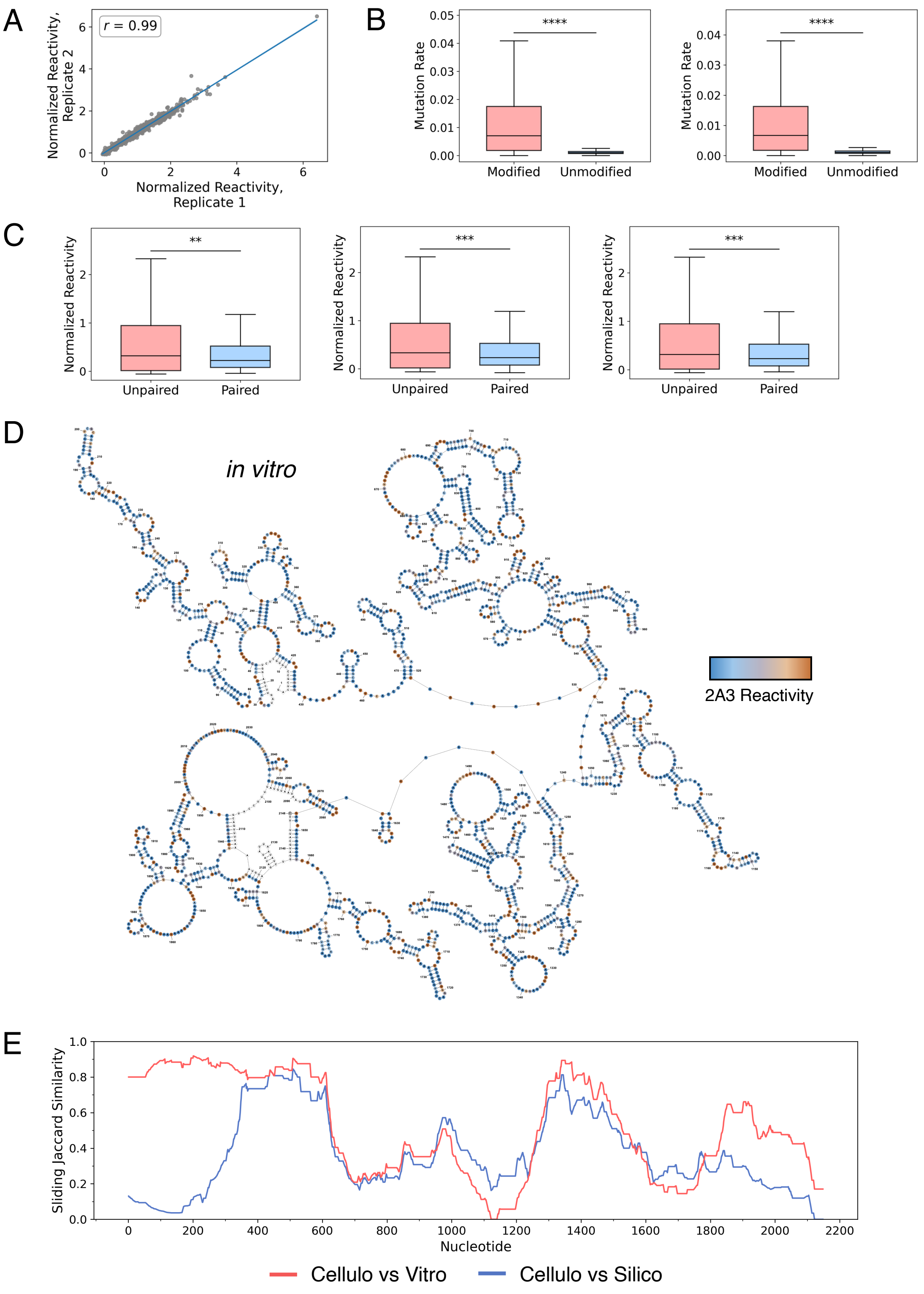

Supplementary Figure S2. Determination of *in vitro* secondary structure of HOTAIR, structured modules *in cellulo*, and comparison to *in silico* prediction. (A) Global correlation of

normalized reactivities between biological replicates of *in vitro* SHAPE-MaP on HOTAIR. The linear regression line is shown with 95% confidence interval shading. *r*, Pearson's correlation coefficient. (B) Mutation rates of 2A3-modified samples compared to DMSO-unmodified controls for two replicates of *in vitro* SHAPE-MaP on HOTAIR. Visualized with a box-and-whisker plot. Statistical significance calculated with Mann-Whitney U test, **** = $p \leq 0.0001$, *** = $p \leq 0.001$, ** = $p \leq 0.01$. (C) Normalized reactivities for residues predicted to be unpaired compared to paired in the secondary structure prediction generated from *in vitro* SHAPE-MaP reactivities of HOTAIR from replicate 1 (left), replicate 2 (middle), and the replicate-averaged values (right). Visualized with a box-and-whisker plot. Statistical significance calculated with Mann-Whitney U test, **** = $p \leq 0.0001$, *** = $p \leq 0.001$, ** = $p \leq 0.01$. (D) Secondary structure prediction of HOTAIR using Superfold with replicate-averaged *in vitro* SHAPE-MaP data. Nucleotides are colored by their normalized reactivity values, orange (≥ 1.0), light orange (≥ 0.75), gray (≥ 0.5), light blue (≥ 0.25), blue (< 0.25), and white (no data). Structure was visualized with StructureEditor. (E) Jaccard similarity comparisons in sliding windows for the *in cellulo*, *in vitro*, and *in silico* secondary structure models of HOTAIR.

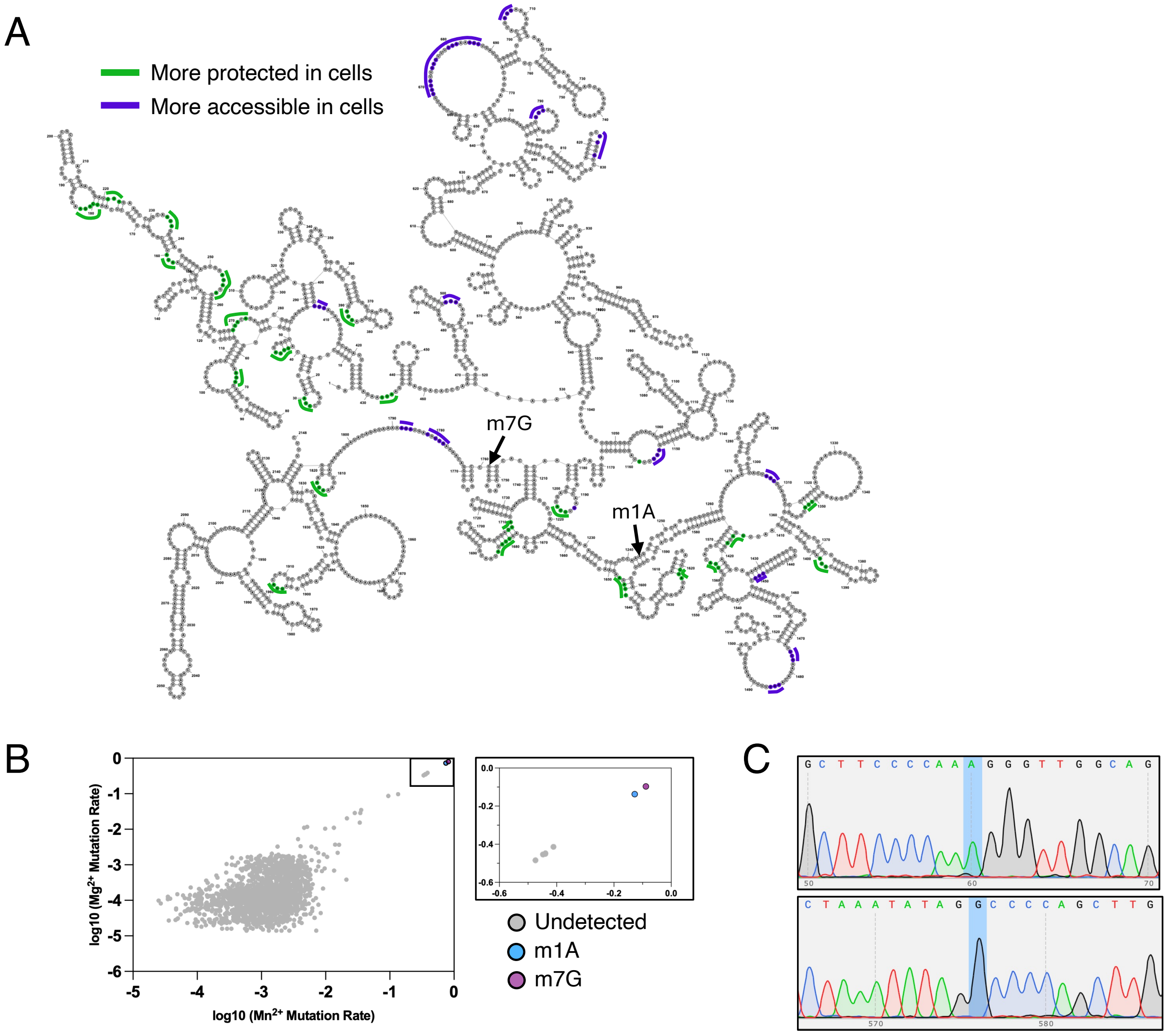

Supplementary Figure S3. Differential reactivity sites and evidence for chemical modifications on *in cellulo* HOTAIR. (A) Structure of full-length HOTAIR *in cellulo* with sites that are more protected *in cellulo* (green, ΔSHAPE > 0) and sites that are more accessible *in cellulo* (purple, ΔSHAPE < 0). Structure was visualized with StructureEditor. (B) Representative replicate from MRT-ModSeq. Plotted in GraphPad Prism. The m1A and m7G modification sites identified here are shown on the structure in (A). (C) Sequencing of genomic DNA for region corresponding to the m1A and m7G sites identified in (B). Visualized in SnapGene.

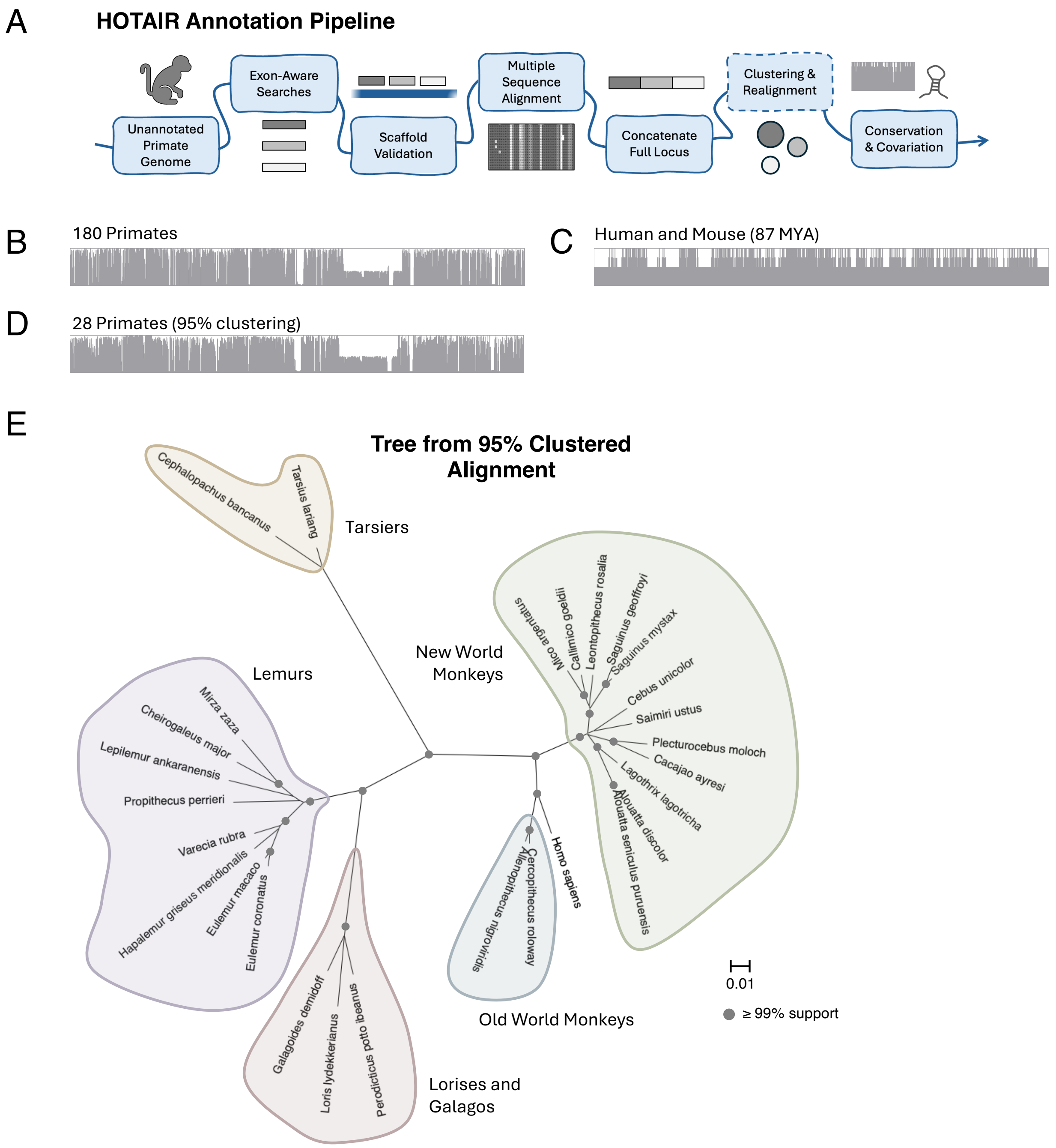

Supplementary Figure S4. HOTAIR sequence annotation and conservation in primates. (A) Pipeline for annotating the HOTAIR locus in primate genomes, building multiple sequence alignments, and analyzing conservation and covariation. (B) Conservation histogram of the alignment of 180 primates, including human. Gray bars represent conservation for each column

in the alignment. Visualized in UGENE. (C) Conservation histogram between human and mouse HOTAIR. Gray bars represent conservation for each column in the alignment. Visualized in UGENE. (D) Conservation histogram of the alignment of 28 primates, including human. Gray bars represent conservation for each column in the alignment. Visualized in UGENE. (E) Phylogenetic tree of primate species calculated from the alignment in (D), where 0.01 = 1% sequence divergence. Tree was built using Maximum Likelihood method and GTR+Γ substitution model in UGENE and visualized in MEGA. Nodes with filled circles represent those with ≥ 99% bootstrap support from 1,000 replicates.

Supplementary Tables

Supplementary Table S1. Domain boundaries for *in cellulo*, *in vitro*, and *in silico* HOTAIR secondary structure models.

Sample	Domain 1 (nt)	Domain 2 (nt)	Domain 3 (nt)	Domain 4 (nt)
Replicate 1, *in cellulo*	1-478	479-1055	1056-1416	1417-2148
Replicate 2, *in cellulo*	1-525	526-1123	1124-1795	1796-2148
Average, *in cellulo*	1-530	531-1046	1047-1747	1748-2148
Average, *in vitro*	1-530	531-1047	1048-1627	1628-2148
Prior structure, *in vitro*[1]	1-530	531-1040	1041-1513	1514-2148
in silico	1-523	524-1122	1123-1780	1781-2148

[1]Somarowthu S, Legiewicz M, Chillon I, Marcia M, Liu F, Pyle AM. 2015. HOTAIR forms an intricate and modular secondary structure. *Mol Cell* **58**: 353-361.

Supplementary Table S2. Conservation of HOTAIR between human and different primate taxa and mouse.

Taxon	Divergence Time from Human (MYA)	Number of Species	Percent Identity to Human	Percent Coverage to Human
Eastern Gorilla	9	1	98.74	99.95
Lesser Apes	19	8	94.81	98.88
Old World Monkeys	29	57	93.30	99.53
New World Monkeys	43	72	76.53	99.10
Tarsiers	69	3	76.37	96.28
Lemurs, Lorises, and Galagos	74	38	78.37	98.09
Mouse	87	1	51.68	83.19

Percent identity and coverage are reported as the median of pairwise identities between each species and human for taxa with more than 1 species. Values for primates were based on a multiple sequence alignment of 180 species.

Supplementary Table S3. Covarying base pairs in the *in cellulo* structure detected in primate alignments of HOTAIR.

**Alignment**	**Clustering (%)**	**Number of Sequences**	**Covarying Pairs in the Structure over Total**	**Covarying Pairs in D1/D2/D3/D4**	**Statistic**	**Window and Slide (nt)**
Full-length	99	60	8/8	0/0/6/2	RAFSp	500/100
Full-length	98	41	1/1	1/0/0/0	RAFSp	500/100
Full-length	95	28	8/8	2/0/6/0	RAFSp	500/100
Domain 3	95	28	8/8	n/a	RAFSp	500/100
Domain 3	95	28	9/12	n/a	RAFSp	300/100
Domain 3	95	28	0	n/a	GTp	500/100
Domain 3	95	28	1/2	n/a	GTp	300/100
